# Vancomycin-lock therapy for prevention of catheter-related bloodstream infection in very low body weight infants

**DOI:** 10.1186/s12887-020-02482-2

**Published:** 2021-01-04

**Authors:** Hong Liang, Lian Zhang, Xiaoping Guo, Li Sun

**Affiliations:** 1 Department of Neonatology, Guangzhou Women and Children’s Medical Center, Guangzhou, 510623 P.R. China; 2 Department of Neonatology, Shenzhen Bao’an Maternal and Child Health Hospital, Shenzhen, 518133 P.R. China

**Keywords:** Preterm infants, Very low body weight, Catheter-related bloodstream infection, Vancomycin, Lock, Prevention

## Abstract

**Background:**

This study was to evaluate the effectiveness and safety of vancomycin- lock therapy for the prevention of catheter-related bloodstream infection (CRBSI) in very low body weight (VLBW) preterm infant patients.

**Methods:**

One hundred and thirty-seven cases of VLBW preterm infants who retained peripherally inserted central catheters (PICCs) were retrospectively reviewed, including 68 treating with heparin plus vancomycin (vancomycin-lock group) and 69 with heparin only (control group). The incidence of CRBSI, related pathogenic bacteria, adverse events during the treatment, complications, antibiotic exposure, PICC usage time, hospital stay, etc. were compared between the above two groups.

**Results:**

The incidence rate of CRBSI in the vancomycin-lock group (4.4%, 3/68) was significantly less than in the control group (21.7%, 15/69, *p* = 0.004). Total antibiotic exposure time during the whole observation period was significantly shorter in the group than in the control group (11.2 ± 10.0 vs 23.6 ± 16.1 d; *p* < 0.001). No hypoglycemia occurred during the locking, and the blood concentrations of vancomycin were not detectable.

**Conclusions:**

Vancomycin-lock may effectively prevent CRBSI in Chinese VLBW preterm infants and reduce the exposure time of antibiotics, without causing obvious side complications.

## Background

Peripherally inserted central catheters (PICCs) are currently commonly used in neonatal intensive care units (NICUs), especially for the treatment of extremely preterm infants and very low birth weight (VLBW) infants. However, usage of PICCs increases the risk of nosocomial infection, documented as catheter-related bloodstream infection (CRBSI) [1–3], which results in extended hospital stay and various life-threatening complications such as necrotizing enterocolitis (NEC), intraventricular hemorrhage (IVH), bronchopulmonary dysplasia (BPD) and retinopathy of prematurity (ROP), leading to a mortality rate of 4–29% [4, 5]. It is vital important to take preventive measures to reduce the incidence of CRBSI. In 2012, a bundle intervention strategy was recommended by the United States Center for Disease Control [6], which showed some efficacy in both adult and pediatric patients [7–9]. However, this strategy has limitations in the treatment for neonate patients, and CRBSI still remains a major and persistent problem in NICU of China’s major medical centers [10, 11].

The pathogenic microorganisms colonized inside catheters can easily form a bacterial biofilm and eventually spread with blood flow [12], which causes CRBSI. Antibiotic-lock therapy (ALT) has been developed that high-dose antibiotic solution dripped and maintained in the catheter cavity for a certain period can dissolve the biofilm formed on the wall to reduce the colonization of the bacteria and kill the embedded bacteria. Viale et al. demonstrated that most (93.3%) patients carrying central line, who received antibiotic locks alone or locks plus systemic antibiotics therapy, successfully retained the central catheter, without the incidence of CRBSI and treatment-related adverse events [13]. A randomized trial showed that catheter locking by taurolidine significantly decreased the incidence rate of CBRSI in children with cancer compared with heparin [14]. Some other studies also reported the benefits from the application of ALT in the prevention of CRBSI [15–17]. However, Megged et al. showed combination of ALT with systemic antibiotics just achieved limited preventive effect on the central venous CRBSI in children [18].

Until now, ALT for neonate patients has been rarely reported [19, 20], and there have no related studies in relation to Chinese preterm infant patients yet. This study was to evaluate the effectiveness and safety of ALT in the treatment of VLBW preterm neonate patients at the NICU of Guangzhou Women and Children’s Medical Center (Guangzhou, China).

## Methods

### Patients

Patients who were admitted to the NICU of Guangzhou Women and Children’s Medical Center between March 2014 and December 2016 were retrospectively reviewed. Inclusion criteria of preterm infants were: 1) patients’ birth weight ≤ 1.8 kg, and required total or partial parenteral nutrition (TPN or PPN), 2) patients received routine retention of PICC line for more than 2 weeks, and 3) patients were not systemically administrated antibiotics during the catheter lock. Patients who: 1) had birth weight > 1.8 kg, 2) died during the first week after birth or died of non-CRBSI reasons, 3) received routine retention of PICC line for less than 2 weeks, 4) were administrated with antibiotics before the catheter lock, 5) required umbilical venous catheters (UVCs), 6) were transferred to other hospitals or departments or failed to complete a clinical observation for at least 2 weeks, or 7) with congenital immunodeficiency, multiple malformations or congenital hypoglycemia, were excluded. The study was approved by the Ethics Committee of Guangzhou Women and Children’s Medical Center (No. 2013–32). Written informed consent was obtained from each infant’s guardians.

### Treatment

Patients received treatment of heparin plus vancomycin (vancomycin-lock group) or heparin only (control group).

Before the lock of PICC catheter, clinicians’ hands and catheter interface were strictly disinfected, and the lock solution was prepared by a qualified staff. Lock was carried out since the first day after the placement of PICC catheter, 3 times daily (q8h). PICC catheter position, redness and swelling, secretion, phlebitis, and obstruction were regularly recorded in a unified and strict way by an independent PICC management team at the NICU according to the centralized management and standard process [6].

In the vancomycin-lock group, 0.5 mL lock solution of 10 IU/mL heparin and 25 μg/mL vancomycin was given. If the disease status required systemic antibiotic treatment, vancomycin-lock was stopped, and only heparin solution would be used instead (q12h).

For each lock, the lock solution was retained for 30 min and was then discarded and flushed away from the catheter with 2 ml of normal saline, followed by addition of the former solution used in the catheter. The lock procedure was carried out 3 times daily (q8h). Blood glucose was measured before and after the lock. Blood samples from the inner PICC catheters and peripheral veins were cultured in a BACT/ALERT 3D automatic blood bacteria culture system (BioMerieux, France). Positive blood samples showing bacteria growth were transferred to blood plates and chocolate plates, and were then cultured in a carbon dioxide incubator at 37 °C for 24 h. The bacteria species were then further identified using a VITEK 2 COMPACT automatic microbial identification and susceptibility system (BioMerieux) and a VITEK MS automatic rapid microbial mass spectrometry detection system (BioMerieux). Cultures that had not been detected bacteria growth for 5 days were considered negative blood culture.

The blood concentrations of vancomycin were measured every 2 weeks. Infection indicators were regularly monitored; once clinical symptoms were observed or infection indicators were elevated, blood from the peripheral vein and inner PICC catheter was cultured immediately. If PICC catheter needed to be removed, the catheter tip was cultured to identify the causative bacteria. At the end of the study, the catheter tip was cultured and the blood vancomycin concentrations were measured.

In the control group, only 0.5 mL 10 IU/mL heparin was used, and most of the other procedures were similar to those in the vancomycin-lock group.

Patients were not administrated with antibiotics before the catheter lock. However, during the treatment if the patients’ symptoms became worse and they were suspected with sepsis, the physicians empirically used antibiotics (such as sulperazone, tienam/mepem, and vancomycin) until a line of indicators excluded the occurrence of sepsis.

During the study period there was no consensus on the selection of heparin- or vancomycin-lock strategy in our center, and physicians themselves decided to choose heparin- or vancomycin-lock. In addition, there was no obvious turning point about the strategy of PICC lock (such as switching from heparin- to vancomycin-lock), although since the late-stage of this study the number of patients received vancomycin-lock increased.

### Measurement

The incidence and onset time of CRBSI were recorded. The pathogenic microorganisms from blood were cultured and identified, and the usage of antibiotics was recorded. The side effects of ALT, such as allergy to vancomycin, influence on body function, incidence of hypoglycemia, PICC fracture, embolism and slippage, and local port abscess during the lock, were observed.

### Definition

CRBSI was determined according to the definition of CRBSI by the United States Center for Disease Control in 2012 [6].

Confirmed CRBSI was determined if: 1) infants exhibited symptoms of sepsis, 2) blood cultures from both the peripheral veins and catheters were identified same types of bacteria, and the catheter blood culture had a positive result 2 h earlier; or the culture of the catheter tip was positive and had the same types of bacteria as the peripheral blood culture, 3) other sources of infection were excluded.

Suspected CRBSI was determined if: 1) there was clinical symptoms of sepsis, 2) peripheral blood culture was positive but without consistent types of the identified bacteria with those from the inner catheter blood or catheter tip, or the culture of the peripheral blood was identified common skin symbionts, such as *Staphylococcus epidermidis*, coagulase-negative *Staphylococcus*, *Bacillus* and *Candida*, 3) bloodstream infection at other lesions were excluded, and antibiotic treatment was needed and clinical symptoms disappeared thereafter.

Suspected infection was defined if there was manifestation of clinical infection or the indicators of inflammation and infection, such as white blood cells (WBC), C-reactive protein (CRP) and procalcitonin, were abnormal, while the culture of blood or catheter tip was negative.

Colonization was defined if the catheter tip culture was positive after the observation, but there were no clinical symptoms in patients and the blood culture was negative. Non-catheter-related infection (i.e. non-CRBSI neonatal sepsis) was mainly determined based on at least 3 of the following criteria [21, 22]: 1) having clinical symptoms of sepsis, such as respiratory distress, apnea, hyperglycemia, hypoglycemia, vomiting and feeding intolerance, 2) blood cultures from within and outside catheters and those from the catheter tips were negative, 3) abnormal laboratory parameters, such as CRP concentration > 10 mg/L and WBC < 5*10^9^/L, 4) incidence of high risk factors for infection, such as group B *Streptococcus* positive in mothers, chorioamnionitis and premature rupture of membranes (PROM) for over 18 h, and 5) antibiotic treatment was needed, and symptoms disappeared after that.

Infection rate per 1000 catheter-days was defined as the number of the infected cases/ total catheter-days X 1000 ‰. During the treatment if patients needed to be systematically administrated with antibiotics, the systematic administration time of antibiotics was determined as the time (day) of intravenous injection of antibiotics, such as sulperazone, tienam/mepem, and vancomycin, during the whole hospital stay.

### Data collection and statistical analysis

Infants’ basic physiological characteristics (such as gestational age, weight, APGAR score, mechanical ventilation [MV] time), suspected infection, major comorbidity (such as neonatal respiratory distress syndrome [NRDS], patent ductus arteriosus [PDA], and non-catheter-related infection), major complications (such as intracranial infection, intracranial hemorrhage, NEC), death, and all adverse events (including local redness and swelling, PICC embolism, rupture and slipping, allergy, adverse drug reactions, and hypoglycemia) during the usage of PICC were collected. The incidence of CRBSI, identified types of pathogenic microorganisms, usage of antibiotics, parenteral nutrition time, PICC time, hospital stay, and hospitalization expenses were also collected.

Data were expressed as mean ± standard derivation or median ± interquartile range. All the above data were analyzed by a qualified statistician who did not participate in this work and was blinded to the grouping situation using SPSS 23 statistical software (SPSS Inc., Chicago, Illinois, USA). Measurement data were compared between the vancomycin-lock and control groups using t-test. Enumeration data were compared between groups using Pearson Chi-square test or Fisher’s exact test. CRBSI risk ratios (RR) and 95% confidence intervals (CIs) were assessed by COX statistical analysis. Probability of non-CRBSI was evaluated by Kaplan-Meier curve analysis. Comparisons with *p* < 0.05 (two-tailed) were thought to be statistically significant.

## Results

**Fig. 1:**
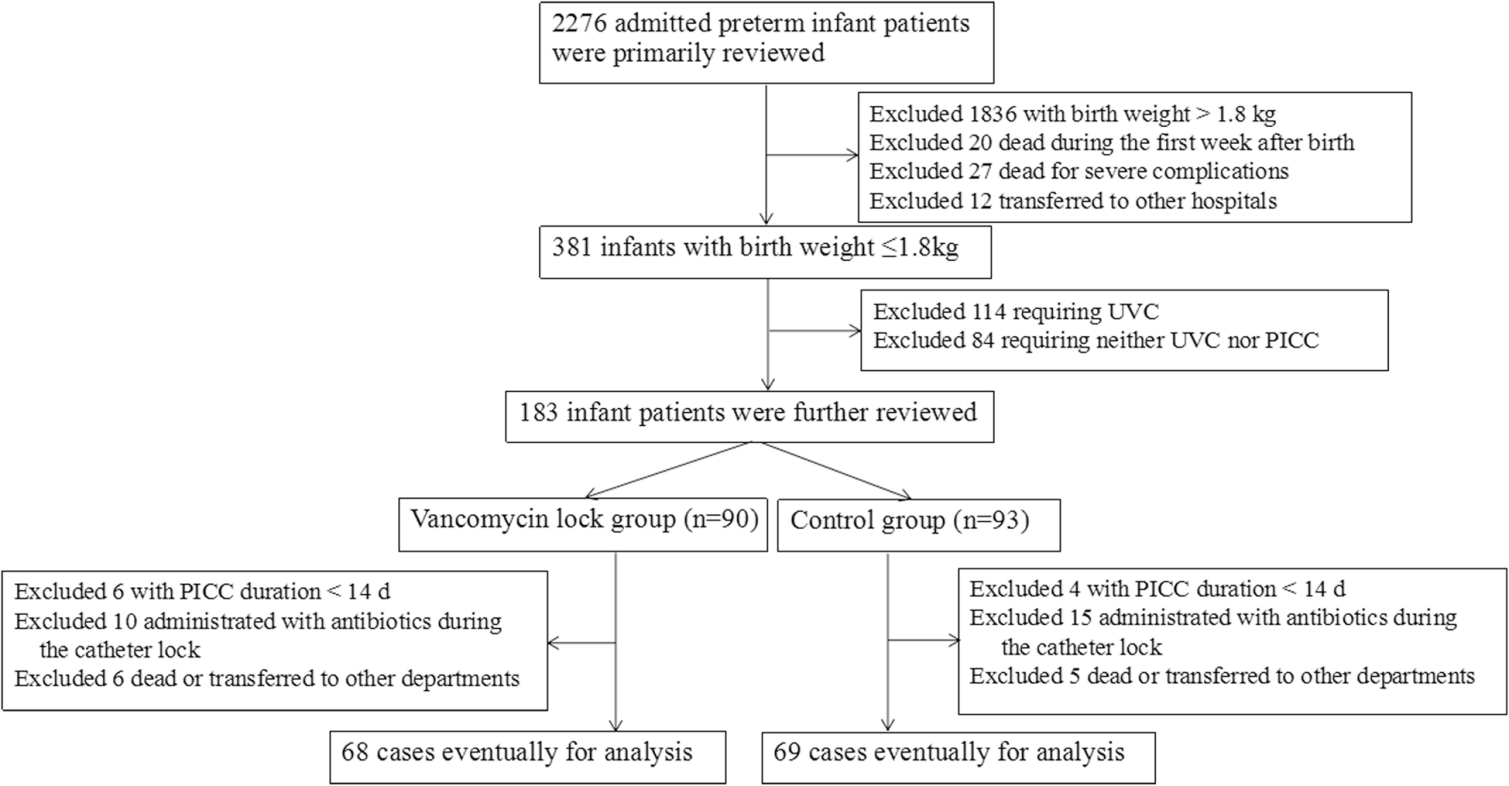
Patient selection flowchart. PICC, peripherally inserted central catheters; UVC, umbilical venous catheters

Two thousand two hundred and seventy-six cases of admitted preterm infant patients were preliminarily reviewed. After exclusion of 1836 cases with birth weight > 1.8 kg, 20 dead during the first week after birth, 27 dead for severe complications, 12 transferred to other hospitals, 114 requiring UVCs, and 84 requiring neither UVC nor PICC, 183 infant patients were further reviewed, including 90 receiving vancomycin-lock (vancomycin-lock group) and 93 receiving heparin only (control group). After another exclusion of cases with PICC duration <14d, those administrated with antibiotics during the catheter lock, and those dead or transferred to other departments, 137 cases were eventually analyzed, 68 in the vancomycin-lock group and 69 in the control group (Fig. 1). It appeared that eventually the numbers of subjects in the vancomycin- and heparin-lock groups were similar, while as a retrospective study, the cases were not randomized to the two groups. Before the exclusion, the numbers of included subjects in the two groups were different although the difference was not too big.

There was no significant difference in gender, gestational age, birth weight, severe comorbidities (including neonatal sepsis, NRDS, NEC, PDA, and IVH above level II), PROM time, MV time, PICC days, TPN or PPN days, hospital stay, and hospitalization costs between the two groups (Table 1). This indicated that the basic information between the two groups were balanced and the comparison between the heparin- and vancomycin-lock related results was meaningful.

**Table 1:** Basic characteristics of infant patients

	Vancomycin-lock group (*n* = 68)	Control group (*n* = 69)	*p* value
Gender (male, n (%))	42 (61.8)	34 (49.3)	0.170*
Gestational age, Wk	29.8 ± 1.90	30.1 ± 2.33	0.323
Birth weight, Kg	1.2 ± 0.19	1.3 ± 0.23	0.765
5-min APGAR score	8.29 ± 1.04	8.7 ± 0.71	0.010
Comorbilities			
Neonatal sepsis, n (%)	43 (63.2)	42 (60.9)	0.861*
NRDS, n (%)	46 (67.6)	44 (63.8)	0.720*
PDA, n (%)	30 (44.1)	31 (44.9)	1.000*
IVH, n (%)	11 (16.2)	16 (23.2)	0.391*
NEC, n (%)	6 (8.8)	7 (10.1)	1.000*
Meningitis, n (%)	4 (5.9)	15 (21.7)	0.012*
PROM time, h	40.2 ± 114	45.3 ± 92.9	0.776
MV time, d	12.8 ± 13.1	17.0 ± 17.9	0.115
PICC time, d	33.0 ± 12.7	33.7 ± 19.6	0.798
TPN or PPN time, d	35.2 ± 15.7	33.9 ± 17.8	0.660
Hospital stay, d	48.3 ± 16.6	51.8 ± 28.2	0.376
Hospitalization costs, RMB (Yuan)	110,000 ± 53,000	118,000 ± 80,000	0.491

Note: *showed chi-square test, and other analysis was t-test; *NRDS* neonatal respiratory distress syndrome, *PDA* patent ductus arteriosus, *IVH* intraventricular hemorrhage, *NEC* necrotizing enterocolitis, *PROM* premature rupture of membrane, *MV* mechanical ventilation, *PICC* peripherally inserted central catheters, *TPN* total parenteral nutrition, *PPN* part parenteral nutrition

### Vancomycin-lock significantly reduced the incidence of CRBSI

As shown in Table 2, the percentage of the total CRBSI cases (including the confirmed and suspected cases) in the vancomycin-lock group (4.4%, 3 of 68) was significantly less than in the control group (21.7%, 15 of 69, RR 0.20, 95% CI 0.06–0.67, *p* = 0.004). Particularly, the percentage of the confirmed CRBSI cases was significantly less in the vancomycin-lock group (4.4%, 3 of 68 vs 15.9%, 11 of 69; RR 0.28, 95% CI 0.08–0.95, *p* = 0.045). The infection rate per 1000 catheter-days was significantly lower in the vancomycin-lock group (1.34‰ in the vancomycin-lock group vs 6.66‰ in the control group, RR 0.23, 95% CI 0.07–0.80, *p* = 0.021).

**Table 2:** Incidence of CRBSI in the vancomycin-lock and control groups

	Vancomycin-lock group (*n* = 68)	Control group (*n* = 69)	RR (95%CI)	*p* value
CRBSI n (%)	3 (4.4%)	15 (21.7%)	0.20 (0.06–0.67)	0.004
Confirmed n (%)	3 (4.4%)	11 (15.9%)	0.28 (0.08–0.95)	0.045
Suspected n (%)	0	4 (5.8%)	0.11 (0.01–2.05)	0.120

Note: CBRSI included the confirmed and suspected cases

Finally, PICC catheter tip culture for bacteria was carried out in the two groups. Bacteria colonization was found in one case of the control group while it was not found in the vancomycin-lock group.

**Fig. 2:**
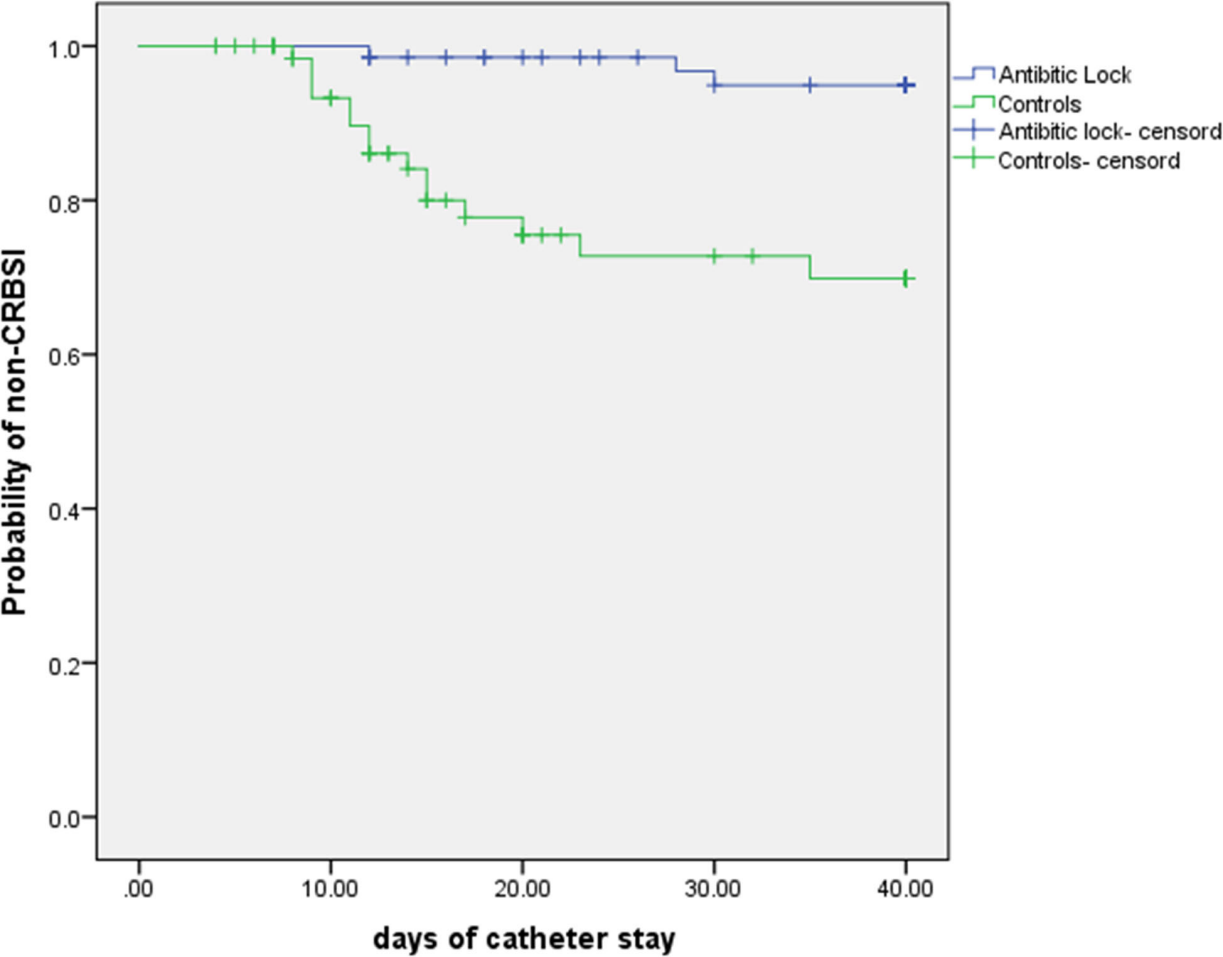
Kaplan-Meier survival curve analysis of the probability of non-CRBSI in the vancomycin-lock and control groups. The probability (log rank (mantel-cox)) of non-CRBSI was significantly larger in the vancomycin-lock group at each same time point after the baseline (i.e. day 10, 20, 30, and 40) (*p* < 0.001). The numbers of patients without CRBSI in the vancomycin-lock and control groups were 68 vs 69 at day 0, 67 vs 54 at day 10, 59 vs 33 at day 20, 53 vs 26 at day 30, and 50 vs 23 at day 40, respectively

Kaplan-Meier survival curve analysis with an observation period of up to 40 days showed that in the same observational time point (i.e. day 10, 20, 30, and 40), the number of patients without CRBSI in the vancomycin-lock group was significantly larger than in the control group (all *p* < 0.001, Fig. 2), indicating a higher probability of non-CRBSI in the vancomycin-lock group. This suggests that vancomycin-lock is beneficial to decrease the incidence of CRBSI and retain the PICC catheters for treatment in NICU.

We then further analyzed the pathogenic bacteria of CRBSI. Table 3 showed 18 patients were infected with pathogenic bacteria, including 12 with Gram-positive bacteria and 6 with Gram-negative bacteria. The Gram-positive bacteria were the major pathogenic microorganisms of CRBSI (66.7%, 12 of 18 cases). In the vancomycin-lock group, only 1 case was infected with Gram-positive bacteria (i.e. *S. aureus*), while in the control group, 11 cases were infected with Gram-positive bacteria, including 2 cases of *S. aureus*, 2 of *Enterococcus faecalis* (including 1 confirmed and 1 suspected cases), 3 of *S. epidermidis*, and 4 of coagulase-negative *Staphylococcus* (including 1 confirmed and 3 suspected cases) (Table 3). The infection rate of Gram-positive bacteria was significantly less in the vancomycin-lock group than in the control group (6%, 1/18, vs 61%, 11/18; *p* = 0.004, Table 3). However, there was no significant difference in the infection rate of Gram-negative bacteria between the two groups (11%, 2/18 in the vancomycin-lock group vs 22%, 4/18 in the control group; *p* = 0.681).

**Table 3:** CRBSI bacteria in the vancomycin-lock and control groups

	Vancomycin-lock group	Control group	*p* value
Gram positive (n/%)	1 (1/18, 6%)	11 (11/18, 61%)	0.004
*S. aureus*	1	2	
*Enterococcus*		2	
*S. epidermidis*		3	
Coagulase negative S*taphylococcus*		4	
Gram negative (n/%)	2 (2/18, 11%)	4 (4/18, 22%)	0.681
*Klebsiella*	1	2	
E. *coli*	1		
*Pseudomonas aeruginosa*			
*Serratia*			
E. *intestinal*		1	
Siamese line well bacteria		1	
*Candida*			
Total	3	15	

### Vancomycin-lock significantly reduced the antibiotic exposure

Generally, in the vancomycin-lock group the systematic administration time of antibiotics was significantly shorter than in the control group (11.2 ± 10.0 vs 23.6 ± 16.1 d, *p* < 0.001, Table 4). For patients with CRBSI, there was no significant difference in the antibiotic treatment time between the vancomycin-lock and control groups (32.7 ± 8.1 vs 31.5 ± 18.0 d, *p* = 0.913). However, for patients without CRBSI, including those with non-catheter-related infection or suspected infection, the antibiotic treatment time was significantly shorter in the vancomycin-lock group than in the control group (10.2 ± 9.0 vs 21.4 ± 11.0 d, *p* < 0.001, Table 4). Therefore, low-dose vancomycin-lock decreases the exposure time of antibiotics in infant patients.

**Table 4:** Treatment time of systematic administration of antibiotics

	Vancomycin-lock group (*n* = 68)	Control group (*n* = 69)	*p* value
Antibiotics covered, d	11.2 ± 10.0	23.6 ± 16.1	< 0.001
CRBSI patients, d	32.7 ± 8.1	31.5 ± 18.0	0.913
Non-CRBSI patients, d	10.2 ± 9.0	21.4 ± 11.0	< 0.001
Non-catheter-related infection patients, d	17.0 ± 7.0	28.8 ± 14.2	0.001
Suspected infection patients, d	7.6 ± 8.3	15.2 ± 12.8	0.008

During the catheter locking period, there were no serious adverse events (such as PICC fracture, slippage and embolism, and local suppuration in the PICC port), no events of allergy to vancomycin, and no incidence of hypoglycemia (< 40 mg/ml). In addition, the serum concentrations of vancomycin both at 2 weeks after the start of catheter lock and at the completion of lock were lower than the minimal detection concentration in plasma (< 3 μg/ml). Therefore, vancomycin-lock did not cause severe adverse events and antibiotic residue.

## Discussion

The commonly used central venous catheterization may lead to CRBSI, a major and persistent problem in NICU of China [10, 11]. ALT has been successfully employed to prevent CRBSI in adults and children patients [15–17, 23–25], while it has rarely been carried out in neonates [19, 20]. In the present study, we evaluated the efficacy of vancomycin-lock technique in the treatment of VLBW preterm infants who needed long-term retention of PICC catheters in NICU. We showed that the CRBSI incidence rate was significantly decreased in the vancomycin-lock group compared with that in the control group, indicating the preventive role of vancomycin-lock in CRBSI. This study could be clinically used to prevent the incidence of CRBSI during the catheter-retaining process in VLBW infants. Until now, there have no similar results in terms of Chinese VLBW preterm infant patients in NICU yet. This study will be beneficial to the application of ALT for VLBW preterm infant patients in not only Chinese populations but also others.

We showed that Gram-positive bacteria were the major pathogenic microorganisms in CRBSI (66.7%). The Gram-positive coccus are the major types of pathogens in bacteria biofilms [26, 27]. Vancomycin has an inhibitory effect on a variety of gram-positive cocci, particularly bacteria in the inner catheter bacteria biofilm [28, 29], and its efficacy and safety in neonates has been confirmed [30, 31]. Therefore, vancomycin was selected for antibiotic-lock so as to prevent the incidence of CRBSI, which is consistent with some reports using vancomycin as the main locking antibiotic [23, 32, 33].

We showed that ALT did effectively reduce the incidence of CRBSI, without obviously causing systemic side effects, which is consistent with Garland et al.’ and Filippi.’ results [19, 20] (Table 5). Interestingly, our result showed a lower infection rate per 1000 catheter-days (1.34‰) in infants receiving ALT when compared with these two studies (2.3‰ and 6.6‰, respectively) [19, 20]. This suggests better efficacy of using ALT to prevent CBRSI in preterm infant patients in our study.

**Table 5:** Comparison of the present study with Galand et al.’ and Filippi et al.’ results

	Galand et al.’ study (Prospective, RCT)	Filippi et al.’ study (Prospective, RCT)	Present study (Retrospective study)
Gestational age, Wk	27 ± 3.8	29 ± 4.8	30 ± 1.9
Birth weight, Kg	1.1 ± 0.72	1.0 ± 0.61	1.3 ± 0.18
PICC time, d	20.3 ± 11.4	7.5 (2–29)	33.0 ± 12.7
CRBSI in AL (n/%)	4.8% (2/42)	6.0% (3/50)	4.4% (3/68)
CRBSI in C (n/%)	30.2% (13/43)	24.5% (13/53)	21.7% (15/69)
*P* value	0.002	< 0.01	0.004

*RCT* randomized controlled trial, *AL* antibiotic-lock group, *C* control treatment without antibiotic-lock. *P* value showed the comparison between the incidence rate of CRBSI in AL and C groups

Although ALT could successfully prevent the incidence of CRBSI, we did not observe significant improvement of treatment outcomes such as MV time, PICC days, TPN or PPN days, and hospital stay. Similarly, in a multicentered (14 NICU centers) prospective study Ruth et al. demonstrated that the outcomes were not obviously improved with the reduction of CRBSI rate [34]. Our and Ruth et al.’s results indicate that contribution of ALT to reduce the incidence of CRBSI is independent of improving the treatment outcomes, which might be related to difference in the types and severity of the diseases in infants.

It is necessary to evaluate the side effects of vancomycin on body. In our study, the blood concentrations of vancomycin were measured every 2 weeks. We showed that all the blood vancomycin concentrations were < 3 μg/ml (lower than the minimum detectable limit). This may be due to that limited vancomycin was locally locked in PICCs during ALT. Given the total administrated dose of vancomycin (3 times) in the catheter was only 75 μg per day and it was eliminated so completely (even undetectable), it would not accumulate in body to bring about side effects.

We demonstrated that vancomycin-lock significantly reduced the antibiotic exposure time during the catheter retaining, which had been rarely reported. Filippi et al. used amoxicillin or gentamicin in all neonate patients for 10 days, fluconazole for within 1 month and sodium fusidate for catheter-locking, and found that the systematic antibiotic exposure time was not obviously reduced with the significant decrease of total CRBSI incidence [20]. In contrast, our ALT procedure appears better. We showed that antibiotic exposure time was generally significantly shorter in patients, particularly in patients with non-CRBSI (regardless of non-catheter-related infection or suspected infection), in the vancomycin-lock group than in the control group. This suggests that due to effectively preventing CRBSI, the local vancomycin-lock in PICCs reduces the requirement of the systematic antibiotic exposure in infant patients, which may decrease the accumulation of antibiotics in body and thus avoid the antibiotic-related side effects. Interestingly, for CRBSI patients, the antibiotic exposure time was insignificantly shorter in the vancomycin-lock group than in the heparin-lock group, while for non-CRBSI patients, the antibiotic exposure time was significantly shorter in the vancomycin-lock group. This may be related to the difference in the response to vancomycin-lock between CRBSI and non-CRBSI patients. The response of non-catheter-related infection (a type of non-CRBSI) to vancomycin-lock appeared to be more sensitive than that of CRBSI, as a result, significantly shorter antibiotic exposure time in the vancomycin-lock vs heparin-lock group was observed in non-catheter-related infection patients when compared with CRBSI patients. For suspected infection (another type of non-CRBSI), antibiotic exposure time was also significantly shorter in the vancomycin-lock vs heparin-lock group, which might be due to that physicians using vancomycin-lock would be more confident to stop the empirical antibiotics usage in time while those using heparin-lock would be more conservative to ensure adequate antibiotics usage. This result needs to be further validated by prospectively randomized controlled studies with larger sample size.

Hypoglycemia is a most concerned side effect during the ALT procedure. The subjects of this study were VLBW preterm infants and they needed continuous infusion of high energy nutrient solution before they could establish intestinal nutrition. During the lock process, we needed to stop the infusion of nutrient solution for 30 min. To prevent the incidence of hypoglycemia, we measured the blood glucose before locking. If the blood glucose concentrations were too low (< 50 mg/dL), the vancomycin-lock procedure was be postponed. Therefore, there were no hypoglycemic events observed.

In our study, vancomycin was used as the locking drug, with strong pertinence and small resistance. Vancomycin-lock could obviously reduce the bloodstream infection of Gram-positive cocci but failed to control that of Gram-negative bacilli. This might be related to its nature as a limited-spectrum antibiotic. Gram-negative bacteria-induced CRBSI may involve a more complex pathogenesis and prevention strategy [35]. Daptomycin has been recently recommended for its strong penetration [33, 36, 37]. Next, vancomycin will be combined with daptomycin or other antibiotics to be used as lock solution for preterm infant patients to achieve better outcomes.

There are several limitations in this study. This was a retrospective study that there might be bias in the selection of cases, due to that subjects were not randomly allocated to receive heparin- or vancomycin-lock, and incompletion of clinical information. In addition, the sample size of this study was not large. A prospectively, multicentered, randomized, controlled, and double-blinded trials with more preterm infant patients are warranted to validate this result.

## Conclusion

ALT with vancomycin is effective and safe in prevention of CRBSI during catheter retaining in Chinese VLBW preterm infant patients. Combination of ALT with other measures, such as bundle management, may further reduce the CRBSI.

## Data Availability

The datasets generated during and/or analyzed during the current study are available from the corresponding author on reasonable request.

## Abbreviations

CRBSI: Catheter-related bloodstream infection
VLBW: Very low body weight
PICC: Peripherally inserted central catheter
NICU: Neonatal intensive care unit
NEC: Necrotizing enterocolitis
IVH: Intraventricular hemorrhage
BPD: Bronchopulmonary dysplasia
ROP: Retinopathy of prematurity
ALT: Antibiotic-lock therapy
TPN: Total parenteral nutrition
PPN: Partial parenteral nutrition
PROM: Premature rupture of membrane
MV: Mechanical ventilation
NRDS: Neonatal respiratory distress syndrome
PDA: Patent ductus arteriosus
WBC: White blood cells
CRP: C-reactive protein
RR: Risk ratios
CI: Confidence intervals
